# How to be a GREAT mentor

**DOI:** 10.1152/advan.00054.2023

**Published:** 2023-06-22

**Authors:** Jamaine S. Davis, Amos M. Sakwe, Aramandla Ramesh, Merry L. Lindsey, Letha Woods

**Affiliations:** ^1^Department of Biochemistry, Cancer Biology, Neuroscience, and Pharmacology, Meharry Medical College, Nashville, Tennessee, United States; ^2^School of Graduate Studies, Meharry Medical College, Nashville, Tennessee, United States; ^3^Research Service, Nashville Veterans Affairs Medical Center, Nashville, Tennessee, United States

**Keywords:** career advice, mentoring, physiology, professional development, training

## Abstract

Formal training in how to mentor is not generally available to students, postdoctoral fellows, or junior faculty. We provide here a framework to develop as a mentor, using the GREAT model. This includes giving opportunities and opening doors; reaching out to help students identify their strengths and reach their goals; encouraging them by serving as a positive example; advising each mentee as an individual; and training them for independent thinking. In this personal view, we expand on each of these steps to illustrate how to develop a personalized mentoring style of your own. By combining these approaches, you as a mentor can work with your mentees to develop an effective and productive mentoring relationship.

**NEW & NOTEWORTHY** We provide here a framework to develop as a mentor, using the GREAT model. This includes giving opportunities and opening doors; reaching out to help students identify their strengths and reach their goals; encouraging them by serving as a positive example; advising each mentee as an individual; and training them for independent thinking.

## INTRODUCTION

In physiology and other biomedical research and education disciplines, a mentor serves to guide their mentees on how to identify and succeed at their career goals (1). Mentors provide career advice and guidance for all steps of career advancement and even beyond. In the research training setting, mentors help their trainees build their knowledge foundation and hone the development of transferrable professional skills (2, 3).

There are different models of mentoring, including the traditional mentor-mentee model where the mentee serves as an apprentice learning from the advisor. Other models include casual or informal mentoring; peer mentoring, where colleagues collaborate to mentor each other; reverse mentoring (also known as managing up), where someone more junior provides guidance and new knowledge to a more senior colleague; shadow mentoring, where a mentor provides dedicated and unbiased mentorship but does not receive any formal recognition for mentoring; and distance monitoring, where mentor and mentee are located in different places and the mentor and the mentee are in touch virtually to render help in the mentee’s personal and career development (4–8).

Any of these models requires intentional mentoring, which refers to mentoring that is deliberate, planned, and purposeful (9). Intentional mentors want their trainees to succeed and work hard to make sure they are not set up to fail (10, 11). They help relieve stress and anxiety by answering questions and providing knowledge and experience, along with reassurance along the way. Mentors help identify opportunities that allow the mentee to grow and develop, even if it means the mentor transfers over an opportunity they were given. For example, an invitation to speak at a national or international conference or an opportunity to peer review could be turned down by the mentor and the mentee could be suggested as a recommended replacement. Mentors also help their mentees figure out best strategies to manage any biases encountered, and this is easier to do the more the mentor resembles the mentee and has shared experiences (12, 13). As one develops a mentoring frame of mind, periodically asking yourself if you are at least minimally providing to the mentee as much as you receive from them in technical support for your research can help you to self-assess how you are doing. Being honest with yourself and your mentee is a requisite for reliable assessment, and the ideal scenario will have both sides benefitting from the relationship.

## MOTIVATORS TO BECOMING A GREAT MENTOR

The reality of mentoring is that not everyone is a great or even good mentor and not everyone has the motivation to develop mentoring skills or even sees the values of mentoring. Themes consistent across outstanding mentors are that *1*) they are enthusiastic and demonstrate strong compassion and selflessness; *2*) they act as career guides; *3*) they commit time and effort; *4*) they support and practice balance; and *5*) they are role models who establish a legacy for others to follow and emulate (14).

Mentoring is not an instinctively useful skill set to every scientist, and mentoring programs are hard to implement and often do not succeed for a number of reasons. One reason is that scientists who are intuitively effective mentors are by nature selfless, which has not historically been viewed as an admirable or productive trait. Another reason why mentoring is sometimes not valued by the scientific community is the perception that effective mentoring takes time, and time to build relationships is viewed as time away from seemingly more important tasks. A further challenge to convincing investigators to enhance their mentoring is that effective mentoring involves use of active listening skills, which again is viewed as unnecessarily time consuming. Other challenges include being comfortable with conflict resolution, knowing when there is a mismatch of mentor and mentee, and a lack of appreciation of mentoring by institutions. In actuality, effective mentoring yields a more efficient process, which can increase research output by reducing the time and effort per experiment. Investigators who do not value the intrinsic importance of mentoring may be attracted to improving with the productivity motivation.

There are many benefits to being an effective mentor, including both direct tangible metrics as well as feel-good mental health benefits. Developing a culture of intentional mentoring allows a better understanding and appreciation of others (11, 15). When the mentor and mentee pairing is effective, productivity for both increases and the relationship is mutually advantageous. Mentees gain confidence, self-efficacy, reduced stress, and more career satisfaction when their activities and decisions are based on strong review and advice provided to them. There is also a link between effective mentorship and time to degree completion for graduate students (16).

One way to assess whether your mentoring is effective is to evaluate the effectiveness of your communication. Frequent miscommunications can serve as a sign that your mentoring skills need appraisal; for example, active listening skills may need to be further honed or more direct language may need to be used. To ensure effective communication, practice reflection (paraphrasing and restating statements made by the person with whom you are speaking): “Are you saying…?” or “What I hear you say is….” This assures the mentee that you are listening and demonstrates that you are sincerely interested in them. Active listening also provides an opportunity to clarify any miscommunication or misunderstandings. When communication is open, this allows the mentor an opportunity to learn from their mentees; if you are not learning, this may mean that your mentees are not comfortable adding their ideas to the discussion.

Regardless of the obvious benefits of effective mentoring, mentor training is not systematically taught or required during training and development, and most mentors end up mentoring along the same style in which they were mentored. In the worst-case scenario, the mentoring style is negative and the mentor is absent, abusive, or unequal in treatment across mentees, whether intentional or not; the mentor does not align with the mentee in terms of personality or ability to provide career and psychosocial support; or the mentor does not share goals or expectations (17). As negative mentoring is a strong impediment to our field by driving out physiologists who would be useful contributors, there is a need to provide guidelines on how to develop the best mentoring strategy for yourself. One could assess their mentoring effectiveness by allowing their mentees to perform a blinded review of them; however, this would require sufficient sample sizing for blinding to actually occur, and action needs to occur as a result. If no changes are made based on the review, future assessments will be meaningless. The aim of this personal view, accordingly, is to provide a structured guide to help mentors assess self-effectiveness and refine as needed.

## USE THE GREAT MODEL TO DEVELOP YOUR MENTORING SKILL SET

The first step in setting up a successful mentor and mentee partnership is to develop a mentoring plan. The best mentoring plans are specific, provide opportunities for the mentee to grow, and use continuous effective communication to provide feedback. The mentoring plan should include activities in which both mentor and mentee will engage so that responsibility is shared and clearly stated. Mapping a mentoring road map involves self-reflection and honesty on the part of the trainee, as well as establishing a trusting environment by the mentor, to establish, maintain, and advance the relationship with both sides committing to the effort (18, 19).

We provide here a template for developing or enhancing mentoring skills, using the GREAT model to *1*) give opportunities and open doors; *2*) reach out to help students identify their strengths and reach their goals; *3*) encourage them by serving as a positive support network; *4*) advise each mentee according to individual personality, career interests, and individual goals; and 5) train them for independent thinking. Developing better mentoring skills will facilitate the development of individual mentee training plans that are realistic and achievable. Table 1 provides examples of how to accomplish each step. Using this template, even seasoned mentors can improve on or hone their abilities to help the next generation of physiologists and other biomedical scientists achieve their career goals.

**Table 1.** The five steps to being a GREAT mentor

GIVE opportunities and open doors	Set up clear expectations from the start (e.g., AAMC compact: https://www.aamc.org/what-we-do/mission-areas/medical-research/grad-compact and https://www.aamc.org/what-we-do/mission-areas/medical-research/post-doc-compact).
	Promote and sponsor your trainees (e.g., recommend for manuscript and grant reviewing or speaker invitations, nominate for awards).
	Teach them professional skills beyond the bench (e.g., networking, writing, and marketing skills).
REACH OUT to help students identify their strengths and reach their goals	Take time to understand each mentee.
	Help them to identify their strengths and weaknesses so they can build on their strengths and minimize their weaknesses.
	Continually offer challenges for growth while keeping a safety net for them.
ENCOURAGE them by serving as a positive example	Discuss what success means to you, and ask your mentee to define for themselves.
	Share your skills, knowledge, expertise, and experiences.
	Teach them to identify bias and know when and how to challenge the process.
ADVISE each mentee as an individual	Treat each trainee as their own individual self, tailoring the mentoring plan for achievement of their goals.
	Involve them in your internal and external collaborations, letting them lead as appropriate.
TRAIN for independent thinking	Help them develop research expertise.
	Give them opportunity to develop and test ideas on their own.
	Ask them to set up, test, and revise their research plans.

### Set the Stage: Find Out What the Mentee Wants

The most important step to developing an effective mentoring relationship is to help the mentee clarify their long-range career goals and needs (9, 20, 21). It is critical for the mentee to feel comfortable to share with you their dreams and goals and the ideas on which these are based. This is easily assessed, as mentees will freely share with you if they trust your support and advice. For a majority of students, this involves taking the time for you both to examine and explore the complex interplay of personal and family histories, motivations and curiosities, and real or perceived structural barriers the mentee is facing. The importance of taking the time to meet with your mentee to discuss their career goals and aspirations cannot be overstated. Giving them time and space is also necessary. It is easy to just jump in and assume that everyone wants to mimic your path, when in reality most if not all of our trainees are interested in paths different from the one we took. Taking time to make sure you are targeting your mentoring to their goals is the critical initial step in the process of becoming a great mentor. For trainees who do not know what they want, allow them the opportunity to figure it out. Sometimes, coaching with the use of active questioning can be used to guide them through this process without telling them what you think they should do. You may need to introduce them to colleagues who are in careers that interest them. Provide them with resources to make an informed decision, including resources for self-assessment so that they can understand themselves, and serve as a sounding board when they want to discuss, without giving any judgment on their decisions. Keep the lines of communication open, so if the mentee discovers new information about themselves or another career, which requires an adjustment to their trajectory, they feel comfortable sharing the new goals and directions with you. You will know that you have done this well when trainees return to you later with appreciation for setting them up for success.

### Rely on Resources: Do Not Reinvent the Wheel

In graduate school and postdoctoral fellowship training, we are not given formal training in mentoring. For those who transition to industry or follow the academic path, there is usually not a formal system in place to develop mentoring skills. For this reason, it is usually left up to the individual to seek training in mentoring. The good news is that there are a number of resources available to help you, in addition to the other references cited in this perspective. Resources range from literature to formal classes to certificate programs in mentoring and coaching. We encourage you not to take on this challenge alone and not rely to only on mentoring as you were mentored. Receiving formal and informal feedback is also important, as is continual self-assessment and adjusting to improve.

Take time for self-reflection and filter the mentoring practices you found to be most beneficial from those that were not instrumental or perhaps were detrimental to your own career growth. Breaking the cycle of bad mentorship starts when one refuses to treat their trainees how they were trained. One way to assess this is to keep track of the trainees who stay in contact with you, as this is a direct reflection of the importance they give to your advice. Likewise, if you had the benefit of an excellent mentor, consider which aspects of their mentoring style you found most effective and would like to emulate. Learning to mentor is like learning to write or teach: the more thinking, planning, and practice you put into it, the more likely you will end up being good at it.

The best mentors provide equal part knowledge and equal part sponsorship to their trainees (1). For research skills, this includes making sure they have the necessary technical and professional skills; have a foundation of knowledge to establish their expertise; know how to design experiments, analyze data, and interpret results; have a strong backbone in research ethics, rigor, and reproducibility; and develop self-confidence in their capabilities (3). Providing them an opportunity to develop self-confidence is the best way to combat against future biases and prejudices they may encounter (22, 23). For career skills, this includes providing guidance on how to actively listen, align expectations, and build trusting collaborations; helping them develop an identity and sense of belonging in the field; and providing practice to understand best ways to diminish effects of bias.

For any successful career, resilience and strong coping mechanisms are requirements and giving positive examples of ways to handle rejection is necessary. As a sponsor, mentors help to foster the independence of their trainees, promote their professional development, teach them to network, and advocate to propel their success. One example of helping your mentees develop resilience is by establishing a team culture of positivity. For manuscript decisions where the journal has left the door open for revision (no matter how major a revision is required), taking a positive tone of congratulating the first author will develop a mindset in them that fosters current and future success. When the manuscript is rejected by the journal, letting them feel bad for a night and then coming in the next day with a plan will allow them to acknowledge the rejection without allowing it to take control. Creating a positive feedback loop through effective mentoring will also promote cultural change in our future leaders, which is a particularly important aspect for creating a more diverse scientific workforce (24, 25). A direct effect of a more diverse scientific workforce is a more productive workforce.

Another component to building resilience and effective coping strategies is to actively celebrate success. As physiologists and biomedical researchers and educators, we are trained to focus on the negative and what needs to be fixed, often glossing over successes as the expected outcome. Taking time to celebrate and acknowledge success will counterbalance the times when rejection is the result of our efforts. Ask yourself periodically when was the last time the team celebrated a success. Providing a positive environment is especially important for women and minority groups underrepresented in science who are already facing systemic biases. Mentors who understand that differences in the system exist can purposefully align their advising strategies to ways that best support the individual student (26, 27). Using the GREAT model, you will be able to develop a mentoring style that works for you and your trainees.

## CONCLUSIONS

In summary, we provide here a framework for developing a plan to develop into a GREAT mentor. As you develop and practice your skills, you will want to periodically self-assess using strategies detailed above. Mentoring success metrics include mentees who attain their milestones, obtain the necessary competencies to define career options and goals, are provided with enough examples to handle future experiences, and are supplied with opportunities that allow them to grow. Basically, the mentee feels prepared for their future if the mentor pairing was successful. Another useful self-assessment for examining how good a mentor you are can be found in Lee et al. (28). This includes assessment of one’s ability to appreciate individual differences; being available; letting mentees self-direct (ranging from totally hands off to micromanaging); using active questioning to coach rather than telling them what to do at every step; celebrating their achievements; providing a laboratory environment of team science and social connection rather than competition; helping them develop skills in research, management, and communication; providing networking opportunities; and serving as a mentor and ultimately a colleague for life.

We hope that you find the GREAT model useful for adopting in your mentoring programs not only in science, technology, engineering, and math (STEM)-related disciplines but also in various health professions programs.

## GRANTS

We acknowledge funding from the Alzheimer’s Association under AD Strategic Fund Award ABA-23–975038 (J.S.D.); from the National Institutes of Health under award numbers SC1GM139814 (A.M.S.) and R25GM116727 (L.W.); from the US Department of Energy under award number DE-EM0005266 (A.R.); and from Biomedical Laboratory Research and Development Service of the Veterans Affairs Office of Research and Development under award number 5I01BX000505 (M.L.L.).

## DISCLAIMERS

The content is solely the responsibility of the authors and does not necessarily represent the official views of any of the funding agencies.

## DISCLOSURES

M. L. Lindsey is editor-in-chief of *American Journal of Physiology-Heart and Circulatory Physiology*. Given her role, she was not involved in the peer review of this article outside of being an author and had no access to information regarding its peer review. None of the other authors has any conflicts of interest, financial or otherwise, to disclose.

## AUTHOR CONTRIBUTIONS

M.L.L. and L.W. conceived and designed research; M.L.L. and L.W. drafted manuscript; J.S.D., A.M.S., A.R., M.L.L., and L.W. edited and revised manuscript; J.S.D., A.M.S., A.R., M.L.L., and L.W. approved final version of manuscript.
